# Automated Segmentation of Lymph Nodes on Neck CT Scans Using Deep Learning

**DOI:** 10.1007/s10278-024-01114-w

**Published:** 2024-06-27

**Authors:** Md Mahfuz Al Hasan, Saba Ghazimoghadam, Padcha Tunlayadechanont, Mohammed Tahsin Mostafiz, Manas Gupta, Antika Roy, Keith Peters, Bruno Hochhegger, Anthony Mancuso, Navid Asadizanjani, Reza Forghani

**Affiliations:** 1 Radiomics and Augmented Intelligence Laboratory (RAIL), Department of Radiology and the Norman Fixel Institute for Neurological Diseases, University of Florida College of Medicine, 1600 SW Archer Road, Gainesville, FL 32610-0374 USA; 2 Department of Radiology, University of Florida College of Medicine, Gainesville, FL USA; 3 Division of Medical Physics, University of Florida College of Medicine, Gainesville, FL USA; 4 Department of Neurology, Division of Movement Disorders, University of Florida College of Medicine, Gainesville, FL USA; 5 Department of Electrical and Computer Engineering, University of Florida College of Medicine, Gainesville, FL USA; 6 Augmented Intelligence and Precision Health Laboratory, Research Institute of the McGill University Health Centre, Montreal, QC Canada; 7 Department of Diagnostic and Therapeutic Radiology and Research, Faculty of Medicine Ramathibodi Hospital, Ratchathewi, Bangkok, Thailand

**Keywords:** Deep learning, Neural networks, Lymph nodes, Lymphadenopathy, Head and neck, Segmentation

## Abstract

Early and accurate detection of cervical lymph nodes is essential for the optimal management and staging of patients with head and neck malignancies. Pilot studies have demonstrated the potential for radiomic and artificial intelligence (AI) approaches in increasing diagnostic accuracy for the detection and classification of lymph nodes, but implementation of many of these approaches in real-world clinical settings would necessitate an automated lymph node segmentation pipeline as a first step. In this study, we aim to develop a non-invasive deep learning (DL) algorithm for detecting and automatically segmenting cervical lymph nodes in 25,119 CT slices from 221 normal neck contrast-enhanced CT scans from patients without head and neck cancer. We focused on the most challenging task of segmentation of small lymph nodes, evaluated multiple architectures, and employed U-Net and our adapted spatial context network to detect and segment small lymph nodes measuring 5–10 mm. The developed algorithm achieved a Dice score of 0.8084, indicating its effectiveness in detecting and segmenting cervical lymph nodes despite their small size. A segmentation framework successful in this task could represent an essential initial block for future algorithms aiming to evaluate small objects such as lymph nodes in different body parts, including small lymph nodes looking normal to the naked human eye but harboring early nodal metastases.

## Introduction

Head and neck cancers comprise a diverse group of tumors that arise from the mucosal surfaces of the oral cavity, pharynx, and larynx in addition to tumors arising in the thyroid gland and salivary glands, among other less common primary tumors arising from other organs in the head and neck [1–3]. Head and neck squamous cell carcinomas (HNSCC) are the most common mucosal malignancies in the head and neck. Identifying and accurately characterizing cervical lymph nodes is essential for the initial staging and surveillance of HNSCC [4]. Determination of the presence and extent of nodal metastases is a requirement for the staging of HNSCC as part of the American Joint Committee on Cancer (AJCC) tumor node metastasis (TNM) classification, and this, in turn, is used to determine the optimal treatment regimen and patient prognosis [5–8].

Imaging plays an integral role in initial tumor staging as well as post-treatment tumor surveillance of HNSCC, enabling confirmation of clinically suspected lymphadenopathy as well as clinically unsuspected pathologic lymph nodes in deep nodal stations not palpable on clinical physical examination [9]. The main anatomic imaging modalities used for the evaluation of cervical lymph nodes are computed tomography (CT), magnetic resonance imaging (MRI), and ultrasound (US) [9, 10]. These studies may be further complemented by functional metabolic imaging techniques such as positron emission tomography (PET). The approach for imaging of the neck will vary based on the suspected primary pathology and to some extent based on institutional variations. However, CT is commonly the first-line imaging modality used for the initial evaluation of most non-thyroid-related head and neck pathologies, and at many institutions, the imaging modality is used for the initial staging of HNSCCs, particularly below the level of the hard palate.

In current clinical practice, evaluation of lymph nodes on CT is based on 2 dimensional measurements and various morphologic criteria evaluated by experts [9]. Despite significant advances in lymph node evaluation and classification, expert classification and discrimination of abnormal from normal lymph nodes are imperfect, and the accuracy can be even less when interpretation is performed by radiologists not subspecialized in head and neck imaging. In particular, detection of early nodal metastases in small lymph nodes measuring less than 1 cm remains a significant challenge [8–11]. As a result, patients having mucosal cancers at certain high-risk sites with greater than 15–20% risk of associated nodal metastases routinely undergo neck dissections, even if this could result in overtreatment of potentially up to 60–70% of patients [5–8, 12]. Even though functional metabolic techniques such as PET scans can increase sensitivity for detection of early nodal metastases, the sensitivity is still not sufficient for precluding elective neck dissections. In addition to limitations in detection of early metastatic nodal spread, current approaches for evaluating lymph nodes in the clinical setting may not enable ready discrimination of the type of pathology involving a lymph node in the absence of a clear primary tumor or other known or identifiable primary etiology.

In addition to a general interest in semi-automation and augmentation of image analysis tasks using artificial intelligence (AI) in diagnostic radiology, there has been a specific interest in using different radiomic or computer vision approaches, including deep learning, for improving accuracy of detection and classification of lymph nodes [13–23]. However, particularly because of the small size of lymph nodes, to be successful, most of the current approaches are contingent first on object (lymph node) identification and segmentation. Automation of these steps would also be a prerequisite for any such tool to be seamlessly integrated into the busy clinical workflow for adoption in clinical practice to impact patient care. Therefore, development of robust, automated, or semi-automated nodal detection and segmentation tools is essential and an important barrier that needs to be overcome in order to effectively deploy these techniques into clinical practice. Automated deep learning segmentation of lymph nodes is uniquely challenging because of the typically small node size occupying a small percentage of pixels on a given scan. This is particularly the case for those nodes where machine-assisted classification is likely to have the greatest impact—i.e., the small nodes measuring less than 1 cm where expert evaluation is least accurate. Outside of diagnostic radiology, organ segmentation is routinely performed in radiation oncology where automated or semi-automated approaches of this variation prone, time-consuming task also has great potential for efficiency and reduced variability [24, 25].

Deep learning techniques have exhibited exceptional performance in computer vision tasks, encompassing semantic segmentation, object detection, and regression prediction, and have become popular for automated segmentation on medical images [25, 26]. Various deep learning architectures, such as U-Net, fully convolutional network (FCN), region-based FCN (R-FCN), generative adversarial network (GAN), and others, have been used for organ segmentation [27]. U-Net, a modified architecture of the fully convolutional network, may require a few annotated images for training while achieving more precise segmentation [28]. Due to its widespread adoption, U-Net variants such as 3D U-Net and DCAN (deep contour-aware networks) have been developed [29, 30]. Recently, Hatamizadeh et al. [31] integrated transformers into the U-Net’s encoder block, improving performance on the Beyond The Cranial Vault (BTCV) dataset.

Self-configuring U-Net architectures (Isensee, 2021 #) have also been developed to address the increasing complexity of deep learning architecture design [32]. GANs and their variants have proven effective in constructing accurate segmentation maps of multiple organs [33]. Semi-supervised learning techniques have been developed to overcome organ segmentation challenges as well [33]. However, despite their success in automated segmentation and detection, deep learning architectures still face challenges in segmenting objects that occupy only a small fraction of pixels in the input volume [34]. Continuous convolution and pooling can distort the input image, losing essential long-range feature information for accurately segmenting small objects. For example, Iuga et al. [35] and Taku et al. [36] achieved notable results in lymph node segmentation, but the detection rate for large lymph nodes was considerably higher than for smaller ones. Nayan et al. [37] addressed the image distortion issue by proposing a modified version of U-Net using bilinear interpolation and total generalized variation, achieving high accuracy on various datasets.

Manjunatha et al. [38] proposed a two-stage approach for CT scans of mediastinal and abdominal LNs using a modified U-Net with ResNet architecture, achieving high sensitivity but increased false positives. For false positive reduction, they used a 3D convolutional neural network classifier in stage II. Cai et al. [39] developed a slice-wise label-map propagation algorithm on RECIST, reaching a mean Dice score of 92% on RECIST slices and 76% on lesion volumes [40]. Tekchandani et al. also addressed the cervical lymph node (CLN) malignancy detection (malignant/benign) in a two-stage manner. In the first stage, authors used an attentional U-Net-like architecture to detect probable CLN patches. In later stage, those patches were classified again with a SENet incorporated Vgg-like network. Another work from same group focused on synthesizing more images from limited train set and then trained an underlying inception network to classify the severity of CLN. One major challenge associated with the lymph node detection task is that lymph nodes occupy a relatively small area within the CT slices, leading to severe data imbalance. Tekchandani et al. addressed the issue by generating additional data through augmentation using generative adversarial networks (GAN) [41, 42]. Ariji et al. [43] created a deep-learning model for automatic segmentation and metastasis detection in cervical lymph nodes. For the segmentation part of the task, they reported an overall recall of 0.735 and they did not report Dice scores. Despite the successful application of deep learning models across various domains, their use in evaluating small lymph nodes in head and neck cancer remains scarce [44, 45].

In this study, we developed a deep learning approach for segmentation of small normal lymph nodes in the neck of healthy individuals. To the best of our knowledge, no studies have implemented a model for the automatic segmentation of small normal cervical LN in healthy individuals and from a technical perspective, these can reasonably be assumed to represent the most challenging task consisting of segmentation of multiple small structures or objects on a given scan, each of which constitute only a minor percentage of pixels on that scan. A segmentation framework successful in this task could represent a foundational block for future algorithms aiming to evaluate all lymph nodes, including small lymph nodes looking normal to the naked human eye but harboring early nodal metastases.

## Methods

### Dataset and Preparation

Institutional review board approval was obtained for this retrospective study. A total of 221 contrast-enhanced CT scans consisting of 25,119 CT slices of the neck were included in this study, harboring normal lymph nodes for development of the segmentation task. The inclusion criteria were as follows: (1) a CT scan of the neck performed with contrast, (2) a scan interpreted as normal or with minor inconsequential incidental findings, and (3) adult patients 18 years or older. Exclusion criteria were as follows: (1) any nodal disease or abnormality on the scans, (2) any known or suspected primary malignancy on the scans, (3) any evidence of significant inflammatory change or abscess on the scans, and (4) any patient history of known malignancy. The dataset was stored as DICOM files and imported into the open-source medical image visualization software, 3D Slicer version 5.0.3.

Cervical LNs are classified into seven anatomical levels based on standard imaging classification initially proposed by Som et al. and subsequently adopted by the AJCC [9]. Levels I to IV, including 1A, 1B, II, III, and IV, have the highest propensity for LN metastasis from HNSCC and therefore were the focus of this study. Normal lymph nodes measuring ≥ 5 mm in their long-axis diameter on the axial image were manually identified and segmented in the axial plane. Because discrimination of small nodes measuring less than 10 mm, especially those approaching 5 mm can be challenging from other structures such as small vessels in a single plane even by experts, the selected nodes and contours were evaluated by scrolling over multiple slices (to help discrimination from small vessels) as well as in the coronal and sagittal planes, to confirm that structures being segmented represent lymph nodes and avoid inadvertent segmentation of potential mimicking structures. Segmentation was initially performed by a trainee (S.G.) and a neuroradiologist (P.T.). A review of all segmentation masks was performed by a fellowship-trained neuroradiologist and head and neck radiologist with over 10 years of clinical practice experience (R.F.), with adjustments made as needed.

### Architecture Selection

The fundamental way of segmentation of an organ is to find a compressed representation of the input by an encoder and then reconstruct the segmentation map from the compressed representation by a decoder. The model is trained by calculating the loss between the output mask and the ground-truth segmentation mask. Following this fundamental structure, U-Net and its variants have been widely used for segmentation in medical imaging (16, 41, 42). We began with a U-Net-based architecture as our baseline. Afterward, we applied our spatial context network (SNet) to leverage the segmentation task.

#### Attentional U-Net

**Fig. 1:**
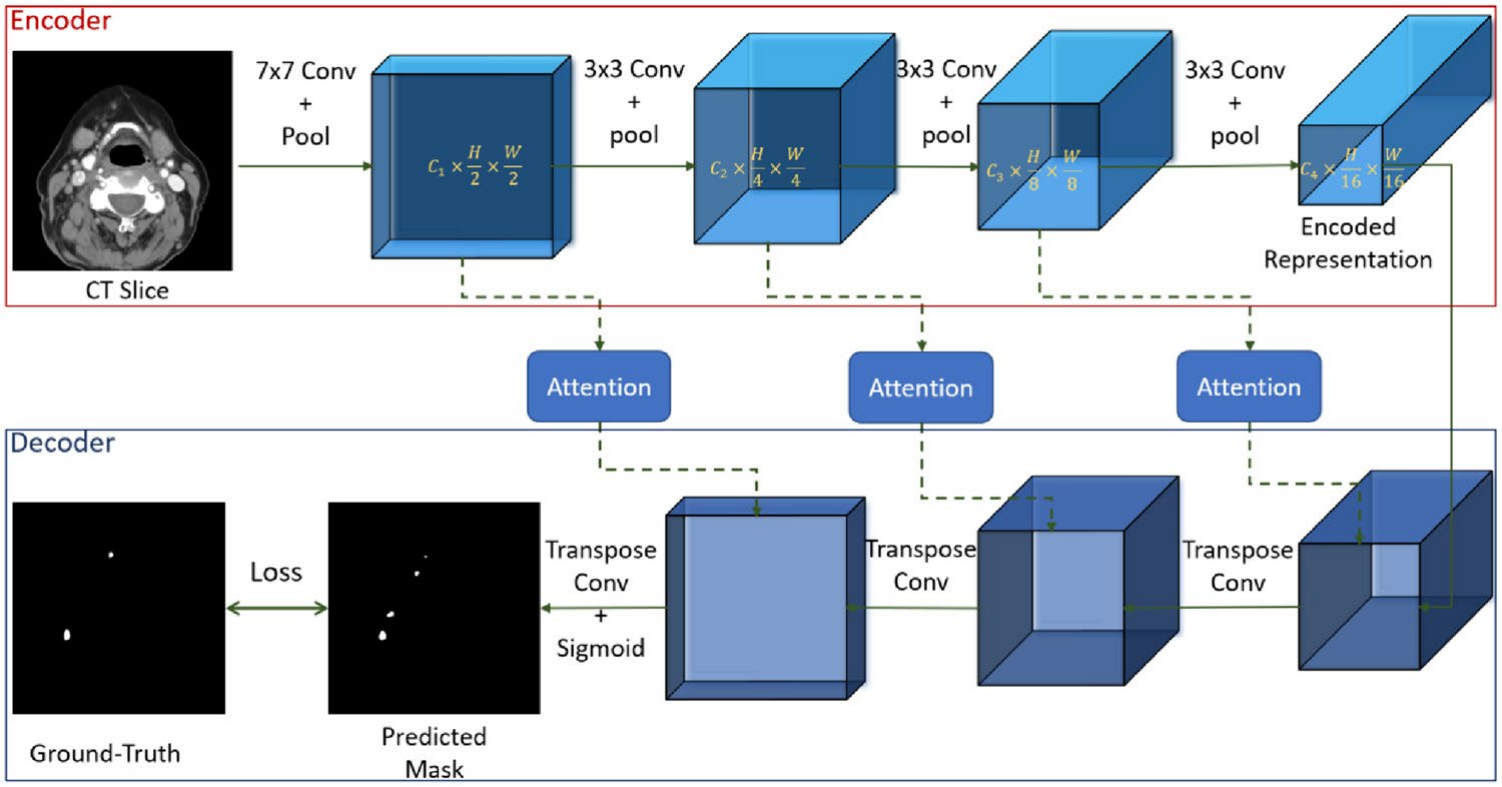
Attentional U-Net architecture for lymph node segmentation. Each convolution (conv) block consists of a batch normalization and an activation unit

**Fig. 2:**
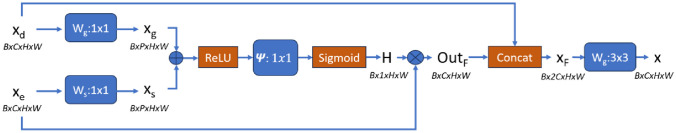
Attention block in attentional U-Net. The decoder (*X*_d_) and encoder (*X*_e_) features are first projected into a *P* (< *C*) dimensional vector *X*_g_ and *X*_s_ using *W*_g_ and *W*_s_ projections, respectively. Afterward, on the combined feature (*X*_g_ + *X*_e_) ReLU activation is applied, and then *Ψ* transformation projected the feature into a vector of channel 1 (*H*) on which sigmoid is applied. This H vector highlights the important contextual feature that is used to filter relevant features from *X*_e_ by applying simple multiplication. Finally, this filtered feature Out_F_ is concatenated with the decoder feature *X*_d_, and then a 3 × 3 convolution is applied to the concatenated feature (*X*_F_) to get the outcome *X* that represents the decoder feature with relevant contextual information from the corresponding encoder layer

Figure 1 shows a schematic diagram of our attentional U-Net. The U-Net is an encoder-decoder architecture where the input image is passed through several convolutional blocks in the encoder to extract a compressed, deep feature representation. In our case, input is passed through four convolutional blocks with max pooling operations to generate the compressed encoded representation as shown in Fig. 1. This encoded representation is then passed through the decoder layers, consisting of three Transpose Convolution blocks. In each step of the decoder, the feature is first upsampled and then concatenated with the relevant contextual features from the corresponding encoder layer in an attentional fusion manner [46] as shown in Fig. 2. This attentional concatenation is different from skip connection in traditional U-Net where the encoder feature is simply concatenated with the decoder feature and fused using convolutions.

One of the limitations of U-Net’s performance in various medical and non-medical segmentation tasks is the excessive input compression from the encoder module (16 times down-sampling in Fig. 1). This significant down-sampling leads to the loss of crucial information required by the decoder, especially for small organs, to generate a precise and accurate segmentation map. Although incorporating attention layers in the skip connections partially addresses this issue, it only provides a partial solution and does not help when organs are too small (high intra-class imbalance in input data). We therefore investigated other architectures that address this limitation.

#### Spatial Context Network

To address the limitation of U-Net-like architecture on small organ detection/segmentation, we developed the spatial context network where the base architecture of the network is adopted from FocusNet [47]. The network is designed considering the small-scale nature of the LN in our dataset as follows.

##### Encoder

**Fig. 3:**
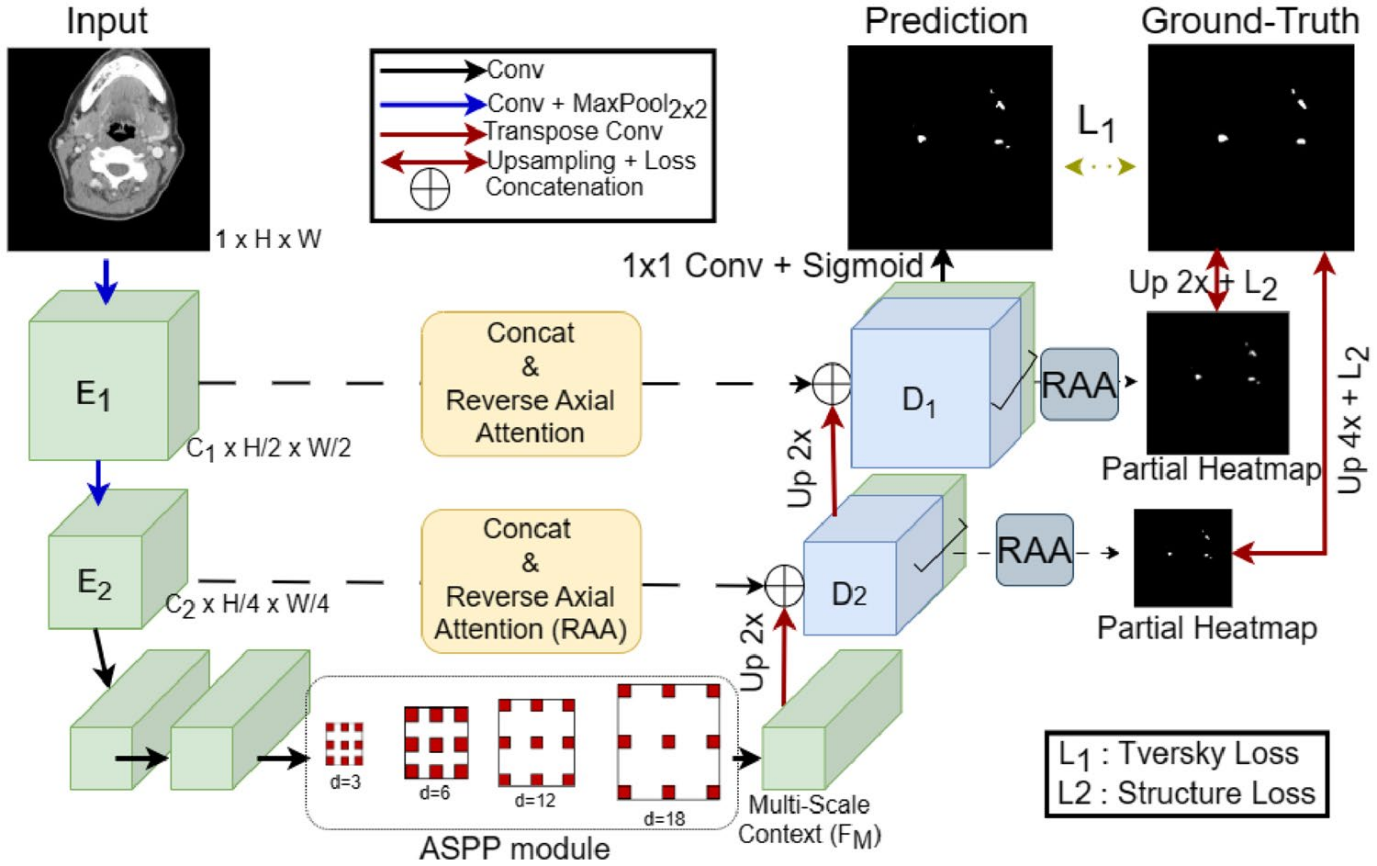
Spatial context network. Atrous spatial pyramid pooling (ASPP) captures multi-scale context from the same feature map

The encoder consists of only 2 down-sampling layers (convolution + max pool) to minimize the information loss as shown in Fig. 3. After that, 2 convolution layers are applied to increase the depth dimension, keeping the same spatial dimension of the feature.

##### Bottleneck Layer

Less down-sampling in the encoder layer results in limited receptive fields, leading to suboptimal global high-level feature learning. To make up for the limited receptive field problem, the following [47] dense atrous spatial pyramid pooling (Dense ASPP) is applied to the encoder features. In ASPP, convolutions with multiple dilation rates are applied to the input feature. This helps capture multi-scale context from the same feature map without down-sampling the original feature map. Output from one dilated convolution (say *d* = 3) gets concatenated with input and then passed through the next dilated convolution layer. The procedure is depicted in Eq. (1) for the first 2 dilation layers of ASPP. Feature processing in the rest two ASPP is like Eq. (1). In our experiments, we used 4 ASPP layers of dilated convolutions with dilations 3, 6, 9, and 12, respectively.

1 $$\begin{array}{l}{p}_{1}{=Conv}_{d=3}\left({e}_{4}\right)\\{p}_{1}=concat\left({p}_{1}, {e}_{4}\right)\\{p}_{2}{=Conv}_{d=6}\left({p}_{1}\right)\\{p}_{2}=concat\left({p}_{2},{p}_{1} \right)\end{array}$$

##### Decoder

Multi-scale context feature (*F*_M_) from ASPP module is passed through 2 decoder layers and the final segmentation output is obtained. First, *F*_M_ is upsampled 2 × using transposed convolution to get the decoder feature (*D*_2_) and gets concatenated with the corresponding encoder feature (*E*_2_) as shown in Fig. 3.

**Fig. 4:**
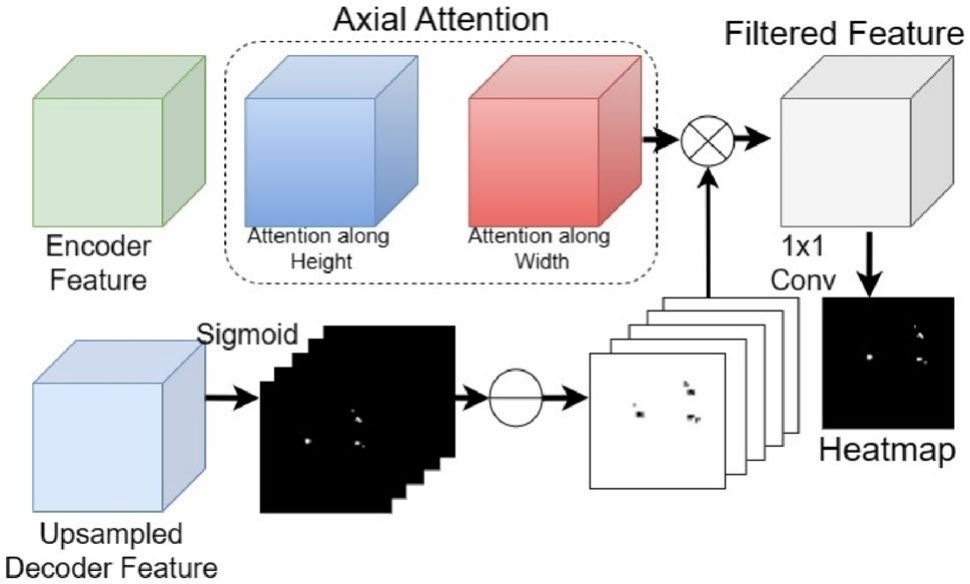
Reverse axial attention in decoder layers

Besides concatenation, a reverse axial attention module is applied between *D*_2_ and *E*_2_ to make the network attend more to the small object region as shown in Fig. 4. Axial attention is applied to the *E*_2_ to obtain the salient information. Axial attention is based on self-attention module which calculates salient information by mapping a query (*Q*) with a set of key-value (*K*, *V*) pairs where *Q*, *K*, and *V* are obtained by taking projections on *E*_2_ as shown in Eq. (2).

2 $$\begin{array}{lll}Q= {Conv}_{Q}\left({E}_{2}\right) & K= {Conv}_{K}\left({E}_{2}\right) & V= {Conv}_{V}\left({E}_{2}\right)\\A=Softmax\left(\frac{Q.{K}^{T}}{\sqrt{{d}_{k}}}\right) & & output=A.V\end{array}$$

The computation cost of self-attention grows quadratically as the spatial size of the input grows. So, following [48], we decomposed 2D attention into two 1D attention along height and width.

For the decoder, we applied sigmoid and then reversed the probability output to detect salient features as shown in Fig. 4. This gets multiplied with the attention output from top branch and finally, we get the context-aware filtered feature. Finally, 1 × 1 conv is applied to the filtered feature to get the heatmap. The heatmap is upsampled and partial loss is calculated with the ground truth as shown in Fig. 3.

Besides RAA, the concatenated feature is passed through 1 × 1 convolution to reduce channel dimension and further upsampled to obtain (*D*_1_) and concatenated with corresponding encoder layer feature (*E*_1_). RAA module is applied here in the same way as before. The concatenated feature map from last layer is passed through 1 × 1 conv and sigmoid to produce the final segmentation output. So, the overall segmentation network is trained by loss between the final segmentation output and ground-truth, and partial heatmap loss from each decoder layer.

## Architecture Implementation and Model Development

### Dataset Split

We collected CT scans of 221 patients. Each CT contains approximately 100 slices on average. 160 out of 221 CT scans were allocated for the training set, the subsequent 40 CT scans for the validation set, and the remaining 21 CT scans for the test set. In total, we had 18,054 CT slices for training, 4463 slices for validation, and 2602 slices for testing the model. Despite working with 2D images, we split the data at the patient level to maintain the integrity of the experiments and avoid data leakage and violation of the independence assumption [49].

### 2D Training

We utilized the S-Net architecture from FocusNet [47] as the backbone of our method. The performance of S-Net is observed by incorporating reverse axial attention tool. The network is finetuned on both classification and localization separately and evaluated. We observed the effect of training and finetuning the network with classification and localization, respectively. As only a few slices contained the lymph nodes in each volume, data was sampled in such a way that during training each batch of data included an equal number of positive (presence of lymph node) and negative (absence of lymph node) 2D slices.

### 2.5D Training

We extended this task to 2.5D training as well. 2.5D training is a hybrid approach that combines elements of both 2D and 3D techniques. In this method, instead of processing each image slice independently (as in 2D), we consider a stack of adjacent slices centered around the target slice. In our setting, we included 7 prior and 7 post slices of a target slice, totaling 15 slices for each data. This allows the model to incorporate some three-dimensional context while still maintaining computational efficiency similar to 2D methods. By analyzing multiple adjacent slices, the model can gain additional spatial information. We used the same set of parameters across 2D and 2.5D training tasks.

### 3D Training

We implemented volumetric 3D training as well. In the case of 3D training, random patches of size 96 × 96 × 96 from the training 3D volumes were extracted and passed through the model. The patch creation was accomplished by the MONAI library. In our implementation, we cropped 2 patches (1 positive and 1 negative) from each CT volume. Evaluation and testing were performed in a sliding window inference manner with a window size of 96 × 96 × 96.

### Augmentation

For both 2D and 2.5D, random rotation (− 10 to + 10°), random vertical flip, random brightness-contrast change, and random gamma transformation were used during training. All of these augmentations were implemented using Albumentations library. For 3D training, random affine transformation is applied with rotation range (0, 0, $\frac{\uppi }{15}$) and scale range (0.1, 0.1, 0.1), and random intensity shift is applied with offset 0.1. For 3D, we used pre-built functions from MONAI library to apply the transformations.

### Training Parameters

A weighted Adam Optimizer with a learning rate of 5e-5 has been used during training in 2D and 2.5D. We kept the learning rate small due to the limited number of lymph node slices available. For 3D, the learning rate was a bit higher (1e-4) as it helped the model to learn faster and better. A batch size of 16 was used for 2D while for 2.5D and 3D, we used batch size of 8. 2D and 2.5D experiments utilized two 12 GB NVIDIA GeForce RTX 2080 GPUs. Later for 3D, we trained the model using an NVIDIA A100 GPU with 80 GB memory.

### Objective Functions

As the targeted object is too small, it is imperative to use an objective function that focuses on the reduction of both false positives (FP) and false negatives (FN) in the prediction. Tversky loss fits perfectly in this scenario which has been used for calculating loss for the final decoder prediction [50].

3 $$\begin{array}{l}Tversk{y}_{index}\left(TI\right)= \frac{TP}{TP+ \alpha *FP+\beta *FN};\\Tversk{y}_{Loss}=1-TI\end{array}$$

To calculate loss on partial heatmaps, weighted IOU (${L}_{IOU}^{W}$) and weighted BCE (${L}_{BCE}^{W}$) loss is used adopted following [48]. ${L}_{IOU}^{W}$ helps learn small organs better by increasing the weights of the foreground pixels to focus it more. ${L}_{BCE}^{W}$ pays more attention to hard pixels like small organ boundaries by assigning more weights to them. We termed ${L}_{IOU}^{W}$ and ${L}_{BCE}^{W}$ as the structure loss. So, the final objective function stands as follows.

4 $$L=Tversk{y}_{Loss}+Structur{e}_{Loss}=Tversk{y}_{Loss}+ {L}_{IOU}^{W}+{L}_{BCE}^{W}$$

### Performance Metrics

For 2D and 2.5D models, performance metric was reported on a per slice basis. Furthermore, the threshold for considering successful prediction of a lymph node on one slice was to have at least 80% overlap per 2D segmentation. As an example, in the case of a lymph node spanning 3 slices, if the segmentation on 2 slices had greater than 80% overlap but on the third slice there was only 60% overlap, the prediction would be 2/3 for that object. For 3D segmentation, the performance metric is reported on a per node/object basis. In addition to the above, Dice score and Intersection of Union were calculated to assess the performance of the segmentation task.

## Results

### Comparison of Baseline Model with Popular Organ Segmentation Networks

We first performed a comparison of our baseline model (w/o attention) with other popular organ segmentation networks (2D) (Table 1). We compared our results with the attention U-Net [51] and CaraNet [48]. This comparison reflects how a lesser amount of downsampling might lead to better segmentation outcomes. We observed a small improvement in performance, measured using Dice score and Jaccard index, using CaraNet compared to attention U-Net (Table 1). However, compared to the network CaraNet [48], there is not a notable improvement (Table 1).

**Table 1:** Quantitative performance

**Network**	**Dice score**	**Jaccard index**
Attention U-Net	0.7513	0.7394
CaraNet	0.7707	0.7602
Spatial context network (w/o attention)	0.7828	0.7740

### Evaluation of Spatial Context Network with Reverse Axial Attention

CaraNet uses reverse axial attention that helps filter out noises outside of the region of interest [48]. Therefore, we evaluated the performance of spatial context network with reverse axial attention on small lymph node segmentation. With the reverse axial attention between encoder and corresponding decoder layers, our model had the best performance compared to the baseline and other popular organ segmentation methods (Table 2).

**Table 2:** Impact of attention module on algorithm performance

**Network**	**Dice score**	**Jaccard index**
Spatial context network (w/o attention)	0.7828	0.7740
Spatial context network (with reverse axial attention)	0.8014	0.78

### Evaluation of Spatial Context Network with Auxiliary Tasks

We next proceeded to evaluate whether an auxiliary task might lead to better context learning. To do this, we adopted the policy of multi-task learning for our framework. For this experiment, it is important to consider two important but interdependent tasks our framework aims to achieve. The first is to detect slices with lymph nodes and second is to localize lymph node regions in those slices.

The first task is to localize the small object, i.e., lymph node. The second is automated contouring of the lymph node, the latter naturally being dependent on successful execution of the first step. Separate experiments were conducted with localization and classification as auxiliary tasks, respectively, and the results presented in Table 3.

**Table 3:** Network performance with auxiliary task

**Network**	**Dice score**	**Jaccard index**
SNEt with localization finetuning	0.8	0.7751
Spatial context network with classification	0.7945	0.7697

From the perspective of performance metrices such as the Dice score or Jaccard Index, we did not observe any improvement in performance (Table 3). However, the network finetuned with localization detected slightly more lymph nodes (Table 4), but an excess of false positives downweighed its performance. On the other hand, the reason that classification hurts the performance is because of the highly imbalanced dataset (too many CT slices with no lymph nodes). In Table 4, we present prediction performance of slice-wise lymph node segmentation.

**Table 4:** Slice level prediction for 2D models

**Network**	**Accurate prediction**	**Misses**
CaraNet	691	669
Our model with attention module	884	476
Ours (with classification)	859	501
Ours (with localization finetuning)	888	472

Prediction and misses are reported based on per slice detection/segmentation of a lymph node with at least 80% overlap per 2D segmentation

### Evaluation of SNet in 2.5D and 3D Training Setup

Our 2.5D spatial context network with reverse axial attention model obtained a Dice score of 0.8011 which is comparable with the performance of the 2D model. On the other hand, the 3D model yielded an inferior result, with a Dice score of 0.68. The potential reasons for this performance discrepancy are explored in the “Discussion” section.

### Qualitative Performance

**Fig. 5:**
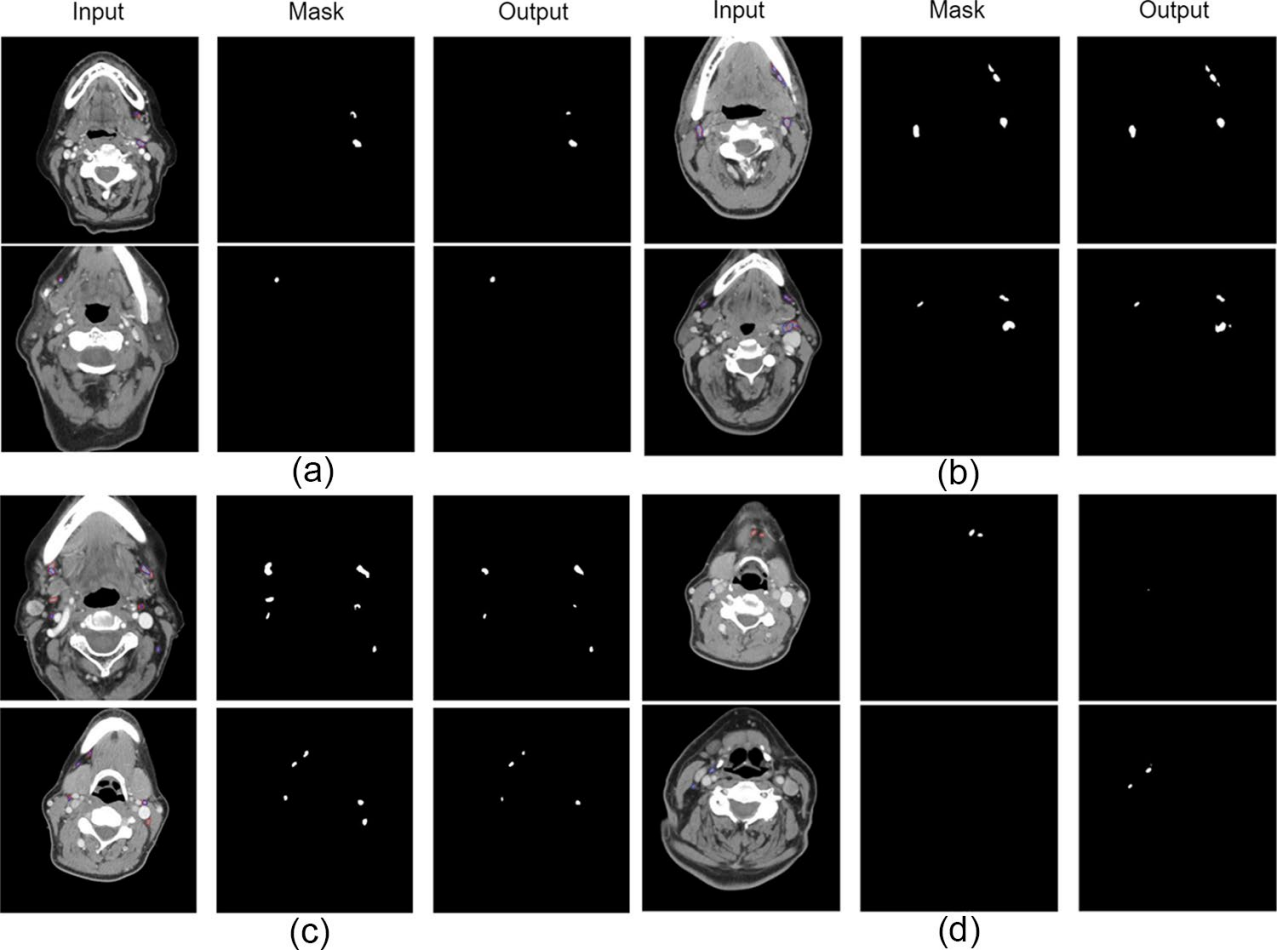
Examples of spatial context network prediction for lymph node segmentation: **a** accurate prediction, **b**, **c** multiple lymph nodes prediction with low false positive, and **d** failed cases. Mask represents the expert-generated contour, and output represents the segmentation by the deep learning algorithm

**Fig. 6:**
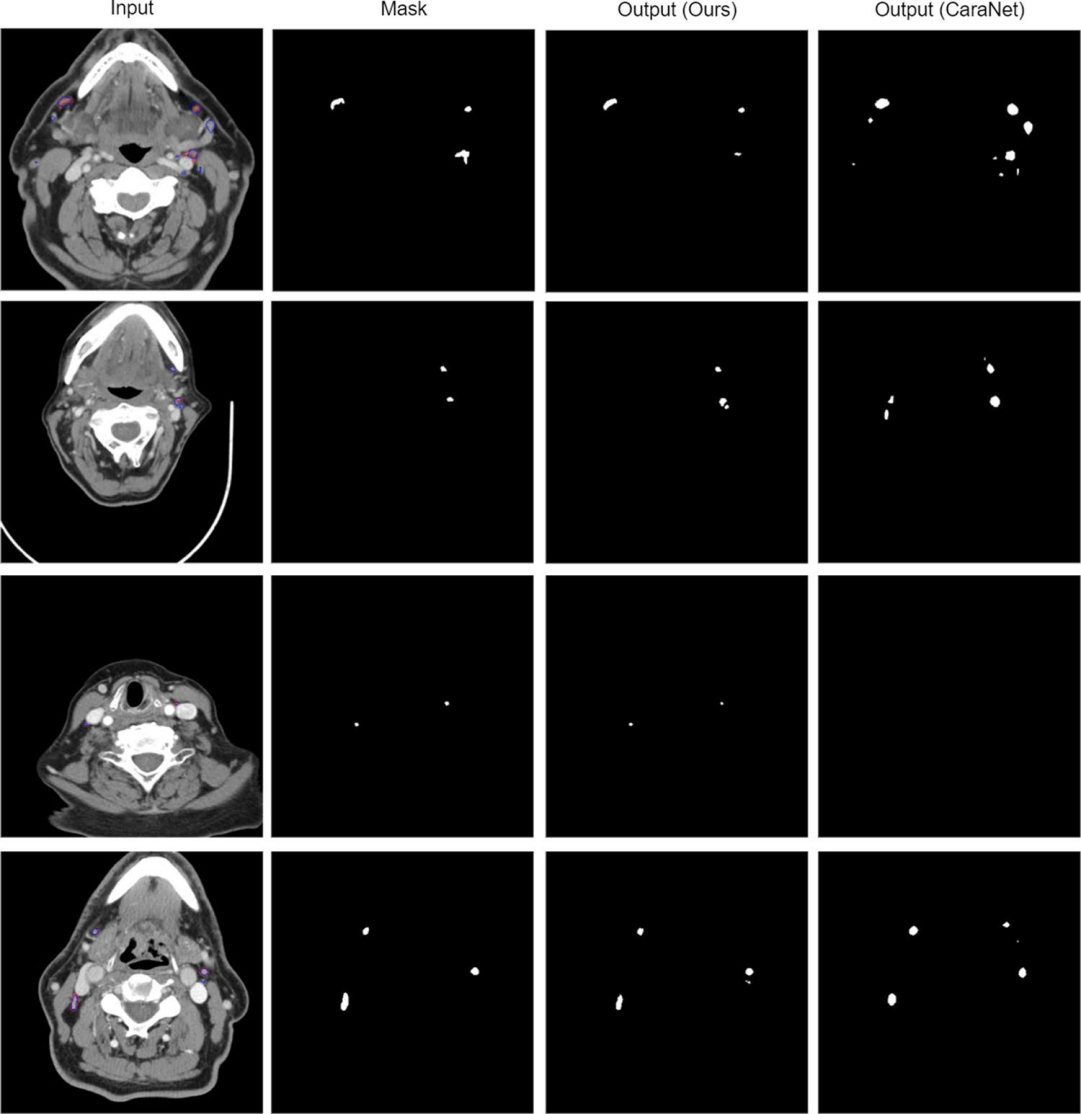
A qualitative comparison between our model and CaraNet. Mask represents the expert-generated contour, and output represents the segmentations by the deep learning algorithm devised in this study (ours) or that using CaraNet

We provide examples of network predictions in Fig. 5. The network demonstrates improved performance even with multiple lymph nodes present (Fig. 5b, c), but sometimes fails to fully detect them (Fig. 5d—first row) or generates false positives (Fig. 5d—second row). A qualitative comparison with the CaraNet outcome is provided in Fig. 6. As demonstrated in Fig. 6, unlike CaraNet, our model is very good at maintaining structural and positional integrity.

## Discussion

Early detection and accurate classification of metastatic pathologic lymph nodes are essential for optimal staging and management of patients with HNSCC. In general, radiologist experts with head and neck expertise can discriminate normal from abnormal lymph nodes with a high degree of accuracy for nodes larger than 1 cm [9–11], although the performance may drop for radiologists who are less familiar or not subspecialty trained in head and neck radiology. On the other hand, for smaller nodes measuring less than 1 cm, the ability of experts in identifying early pathologic lymph nodes decreases, with significant patient care implications. Pilot studies using radiomics and AI suggest that these approaches may be to enhance diagnosis and augment the expert interpretation, potentially enabling identification of early nodal metastases that is undetectable by the naked human eye [13–23]. However, these approaches typically require segmentation of target lymph nodes prior to classification, a task that would be prohibitively time consuming and impractical in clinical practice. An approach enabling automatic detection and segmentation of small objects such as lymph nodes has unique challenges but if solved has applications beyond lymph nodes in the neck, including to other body areas. The aim of this study was to address this initial, fundamental step. To this aim, we developed and assessed a novel deep convolutional neural network algorithm for automatically segmenting cervical lymph nodes, with a good performance resulting in a Dice score of 0.8084 considering the complexity of the task.

We used Dice score and the Jaccard index as the primary measurements used to evaluate performance in this study, the most used metrics for evaluation of segmentation performance. Our study demonstrates that the automatic cervical lymph node segmentation using the proposed approach exhibits strong agreement with expert manual segmentations. Although there is certainly room for improvement from a Dice score of 0.8084, it is worth noting the following. Both Dice score and Jaccard index reflect the performance of the model at the pixel level. However, for small organs like lymph nodes, these measures may not provide a holistic view, since even a small difference that may fall within acceptable variation by experts will be amplified due to the small denominator. Conversely stated, using Dice score to report segmentation of a large organ may provide falsely positive or misleading results, because discordance of a small potentially clinically important will be diluted because of the large denominator [49]. In that regard, although imperfect, we will believe the algorithm’s performance is quite good considering the task of multiple small object segmetnation and nature of the metrics used. In order to capture the multi-scale context of cervical lymph nodes, which are small but vary in scale, our model incorporates ASPP. While this does not fully resolve the issue, it significantly improves upon traditional methods.

Automatic segmentation of lymph nodes consists of two separate but interdependent tasks. The first is to localize the lymph node and the second is automated contouring of the lymph node, the latter step being dependent on successful execution of the first step. We evaluated whether an auxiliary task might help improve learning of the network and used multi-task learning for our framework. This did not improve the Dice score or the Jaccard index. On the other hand, the network finetuned with localization detected slightly more lymph nodes, with the caveat that an excess of false positives downweighed its performance. One reason that the classification hurts the performance is because of the highly imbalanced dataset (too many CT slices with no lymph nodes). As shown in Table 4, our adopted model has outperformed traditional CaraNet by a significant margin in prediction accuracy. However, for most of our models, we have had moderately high false positives which is one of the main challenges in small organ segmentation. When considering the challenges related to false positive classifications in this context, the primary drawback for our model appears to be the inability to distinguish lymph nodes from closely resembling surrounding structures, including small vessels on a slice that can mimic lymph nodes on a single axial slice. This challenge is not unique to machines—when it comes to the evaluation of small lymph nodes, discrimination of small vessels can also be a challenge for experts. In such cases, the expert typically scrolls on multiple slices, and in different planes, to try to make the distinction. In the case of small object detection algorithms, this can be associated with multiple false positive predictions. We are currently working on a more robust small organ detection architecture. In the future, we also intend to investigate a localization assistant network with a classification branch trained in curriculum learning fashion, i.e., using localization as intermediate output to filter out false detections by classification of potential mimicking structures, as one potential option to explore to boost performance. We discuss this briefly in Future Works.

In this work, we evaluated 2D, 2.5D, and 3D architectures. The 2.5D model achieved a similar performance compared to the 2D model. As mentioned in the earlier section, the 3D model resulted in inferior performance. During 3D training, an equal number of volume patches containing positive and negative data were sampled for each batch to ensure that our model learns to differentiate among them properly. It should be noted that small LNs segmented in this study frequently span no more than a few slices. So, when we were cropping volume patches of 96 × 96 × 96, most of the slices from positive volume did not have any LN present in them. As a result, providing the contextual information from the temporal dimension, which is the main advantage of using a 3D model, did not help much in our case. The model size grew larger to fit the 3D data which led to overfitting in our case.

It is also notable to mention that loss functions for 2D were adapted for 3D. However, there is a possibility that the loss function originally designed for the 2D segmentation task is not better suited for 3D segmentation task as they might not be able to enforce sufficient contextual information flow from the temporal dimension. Unlike most work on small organ segmentation, our training setup consists of single-stage end-to-end training. So, the model needs to localize and segment concurrently in a single-stage training which is challenging.

Also, it is hard to separate those small lymph nodes from surrounding soft tissues. Negative patch volume in 3D most of the time resembles closely to the positive patch volume and this makes the learning harder for the model. We are going to investigate issues related to temporal context capturing and separation of similar positive–negative patch volumes to develop a better 3D model in the future. This project has multiple limitations. The dataset used is from a single center, and the results will require validation in larger studies that include data from other centers. For this study, we focused on nodal stations I–IV which are the most involved with mucosal HNSCCs below the level of the hard palate. However, in the future, there would be an interest in developing an algorithm that evaluates all nodal stations in the neck. Lastly, future work focusing on additional optimization using 3D approaches would be of interest.

## Conclusion and Future Work

In this study, we presented a non-invasive deep-learning approach for the identification and segmentation of cervical lymph nodes at levels I to IV on contrast-enhanced CT images. Our model’s performance indicates the potential of deep learning methods for detecting and segmenting cervical lymph nodes. This research serves as the initial step in a broader endeavor to establish the foundation for future radiomic feature extraction from lymph nodes to aid in diagnostic classification of abnormal lymph nodes. Although performed in the neck, the challenging task of small object detection/segmentation is also applicable to other body areas.

While our proposed lymph node segmentation method achieves significant results, its performance is constrained by several factors. Firstly, the current model operates on 2D CT slices; however, incorporating 3D voxel information may provide better context. As part of our future work, we plan to train the model using 3D voxels. Secondly, we applied ASPP to capture multi-context features without excessive downsampling. However, since most lymph nodes are small in scale, a more focused local region-based approach is needed to capture the context in this size range. Lastly, the model is affected by substantial intra-class imbalance (a few pixels in a slice represent lymph nodes, while the majority are background) and inter-class imbalance (more than 70% of CT slices lack lymph nodes). In future work, we plan to develop an objective function that operates in a local patch-based manner, placing more importance on lymph node regions.
